# Author Correction: Cell segmentation-free inference of cell types from in situ transcriptomics data

**DOI:** 10.1038/s41467-021-24405-0

**Published:** 2021-06-28

**Authors:** Jeongbin Park, Wonyl Choi, Sebastian Tiesmeyer, Brian Long, Lars E. Borm, Emma Garren, Thuc Nghi Nguyen, Bosiljka Tasic, Simone Codeluppi, Tobias Graf, Matthias Schlesner, Oliver Stegle, Roland Eils, Naveed Ishaque

**Affiliations:** 1 Berlin Institute of Health at Charité—Universitätsmedizin Berlin, Digital Health Center, Kapelle-Ufer 2, 10117 Berlin, Germany; 2 Faculty of Biosciences, Heidelberg University, Heidelberg, Germany; 3 Division of Computational Genomics and System Genetics, German Cancer Research Center (DKFZ), Heidelberg, Germany; 4 Department of Computer Science, Boston University, Boston, MA USA; 5 Allen Institute for Brain Science, Seattle, WA USA; 6 Division of molecular neurobiology, Department of medical biochemistry and biophysics, Karolinska Institutet, Stockholm, Sweden; 7 Bioinformatics and Omics Data Analytics, German Cancer Research Center (DKFZ), Heidelberg, Germany; 8 Genome Biology Unit, European Molecular Biology Laboratory (EMBL), Heidelberg, Germany; 9 Health Data Science Unit, Heidelberg University Hospital, Heidelberg, Germany

**Keywords:** Gene expression analysis, Fluorescence in situ hybridization, Image processing

Correction to: *Nature Communications* 10.1038/s41467-021-23807-4, published online 10 June 2021.

The original version of this Article omitted Wonyl Choi as an equally contributing author and Roland Eils and Naveed Ishaque as co-supervising authors. This has now been corrected in both the HTML and PDF versions of the Article.

