# Supplementary material for: Cell segmentation-free inference of cell types from in situ transcriptomics data
Source: Nat Commun. 2021 Jun 10;12:3545. doi: 10.1038/s41467-021-23807-4 (PMC8192952; doi:10.1038/s41467-021-23807-4)
Supplement: Supplementary file 2 — Description of Additional Supplementary Files [file 41467_2021_23807_MOESM2_ESM.pdf]

Description of Additional Supplementary Files

File Name: Supplementary Data 1
Description: Primers used for smFISH profiling of the mouse VISp

File Name: Supplementary Movie 1
Description: MERFISH 3D cell-type map, turntable rotating

File Name: Supplementary Movie 2
Description: MERFISH 3D cell-type map, sweeping along z axis by 1 µm

File Name: Supplementary Movie 3
Description: MERFISH neuronal cells, sweeping along z axis by 1 µm

File Name: Supplementary Movie 4
Description: MERFISH astrocytes, sweeping along z axis by 1 µm
