# Supplementary material for: Cell segmentation-free inference of cell types from in situ transcriptomics data
Source: Nat Commun. 2021 Jun 10;12:3545. doi: 10.1038/s41467-021-23807-4 (PMC8192952; doi:10.1038/s41467-021-23807-4)
Supplement: Supplementary file 8 — Reporting Summary [file 41467_2021_23807_MOESM8_ESM.pdf]

nature research

Corresponding author(s): Roland Eils, Naveed Ishaque

Last updated by author(s): Apr 28, 2021

Reporting Summary

Nature Research wishes to improve the reproducibility of the work that we publish. This form provides structure for consistency and transparency in reporting. For further information on Nature Research policies, see Authors & Referees and the Editorial Policy Checklist.

Statistics

For all statistical analyses, confirm that the following items are present in the figure legend, table legend, main text, or Methods section.

n/a	Confirmed	
☐	☒	The exact sample size (*n*) for each experimental group/condition, given as a discrete number and unit of measurement
☒	☐	A statement on whether measurements were taken from distinct samples or whether the same sample was measured repeatedly
☐	☒	The statistical test(s) used AND whether they are one- or two-sided<br>*Only common tests should be described solely by name; describe more complex techniques in the Methods section.*
☒	☐	A description of all covariates tested
☐	☒	A description of any assumptions or corrections, such as tests of normality and adjustment for multiple comparisons
☐	☒	A full description of the statistical parameters including central tendency (e.g. means) or other basic estimates (e.g. regression coefficient) AND variation (e.g. standard deviation) or associated estimates of uncertainty (e.g. confidence intervals)
☐	☒	For null hypothesis testing, the test statistic (e.g. *F*, *t*, *r*) with confidence intervals, effect sizes, degrees of freedom and *P* value noted<br>*Give P values as exact values whenever suitable.*
☒	☐	For Bayesian analysis, information on the choice of priors and Markov chain Monte Carlo settings
☐	☒	For hierarchical and complex designs, identification of the appropriate level for tests and full reporting of outcomes
☐	☒	Estimates of effect sizes (e.g. Cohen's *d*, Pearson's *r*), indicating how they were calculated

Our web collection on statistics for biologists contains articles on many of the points above.

Software and code

Policy information about availability of computer code

Data collection	A Jupyter notebook (https://github.com/HiDiHlabs/ssam_example) outlines the commands used to download and pre-process the data.
Data analysis	The majority of analysis was performed using Python version 3.7.0. The following python packages were used:<br>networkX 2.3, numpy 1.17.1, scipy 1.3.1, pandas 0.25.1, matplotlib 3.1.0, seaborn 0.9.0, scikit-learn 0.21.3, umap-learn 0.3.10, python-louvain 0.13, sparse 0.8.0, scikit-image 0.15.0, pyarrow 0.15.1, packaging 20.8.<br><br>For normalization and variance stabilization of data R v3.6.0 was used with the sctransform 0.2.0 library, requiring feather 0.3.3.<br><br>Plotting was performed using the python packages Mh5py 2.10.0, loompy 3.0.1, matplotlib 3.1.049, matplotlib-scalebar 0.6.0, and Sseaborn 0.9.050, were used to draw 2D images, plots, and heatmaps. We include helper functions in SSAM to easily generate plots.<br><br>For 3D reconstruction the PoissonRecon command-line utility from Adaptive Multigrid Solvers 12.00, Meshlab 2016.12, vtk 8.1.2, and python-plyfile 0.7.1 were used. Supplementary movies were generated by using Virtualdub (1.10.4-AMD64, http://www.virtualdub.org/) and ffmpeg 3.4.4.<br><br>The SSAM tool is available via github (https://github.com/HiDiHlabs/ssam), and the version used for the study (SSAM 1.0.2) is also deposited on Zenodo with a DOI (http://doi.org/10.5281/zenodo.4721907).<br><br>A Jupyter notebook (https://github.com/HiDiHlabs/ssam_example) outlines the commands used to to reproduce the data analysis presented in this study.

For manuscripts utilizing custom algorithms or software that are central to the research but not yet described in published literature, software must be made available to editors/reviewers. We strongly encourage code deposition in a community repository (e.g. GitHub). See the Nature Research guidelines for submitting code & software for further information.

Data

Policy information about availability of data

All manuscripts must include a data availability statement. This statement should provide the following information, where applicable:

- Accession codes, unique identifiers, or web links for publicly available datasets
- A list of figures that have associated raw data
- A description of any restrictions on data availability

The datasets analysed during the current study are available in the Zenodo repository, http://doi.org/10.5281/zenodo.3478502.

Original mRNA spot data is available from:
- osmFISH SSp (Codeluppi et al., 2018) http://linnarssonlab.org/osmFISH/availability/
- MERFISH POA (Moffitt et al. 2018) https://datadryad.org/stash/dataset/doi:10.5061/dryad.8t8s248
- VISp smFISH was generated as part of this study and available via Zenodo, http://doi.org/10.5281/zenodo.3478502

Single cell RNAseq data is available from:
- scRNAseq of SSp for SSAM guided mode analysis (Marques et al., 2016; Zeisel et al., 2015) http://loom.linnarssonlab.org/
- scRNAseq for comparison of total mRNA counts (Zeisel et al., 2018) http://mousebrain.org/.

The high resolution images are available from:
- smFISH VISP is available in star-fish format from https://s3.amazonaws.com/starfish.data.spacetx/smFISH/mouse/formatted_with_DAPI/experiment.json
- osmFISH SSp high resolution poly-A and DAPI images (Codeluppi et al., 2018) are available on request from Sten Linnarsson

Field-specific reporting

Please select the one below that is the best fit for your research. If you are not sure, read the appropriate sections before making your selection.

☒ Life sciences ☐ Behavioural & social sciences ☐ Ecological, evolutionary & environmental sciences

For a reference copy of the document with all sections, see nature.com/documents/nr-reporting-summary-flat.pdf

Life sciences study design

All studies must disclose on these points even when the disclosure is negative.

Sample size	This study was a proof of concept for a new data processing paradigm for spatially resolved transcriptomics data. We demonstrate the tool on 3 high quality datasets - 2 that were previously analysed, and 1 novel dataset. All data analysis was performed in collaboration with the providers of the data (Lars Borm and Simone Codeluppi for osmFISH SSp, Jeff Moffitt for MERFISH POA, Brian Long, Thuc Nguyen and Bosiljka Tasic for the smFISH VISp.)
Data exclusions	No data was excluded.
Replication	To address reproducibility we applied our algorithm to 3 different datasets, of which 2 were previously analyzed. Each dataset was independently generated, from different tissues, and using different imaging techniques. Repeat analysis was successful without modifying parameters.
Randomization	Randomization is not relevant for this study. Instead, parameter estimation was relevant (we investigated the choice of major parameters, demonstrated in supplementary figures), and found that a particular parameter set was appropriate for multiple datasets, and as such were chosen and default parameters.
Blinding	Blinding was implemented through use of unsupervised analysis using SSAM, with no effort to replicate the exact results of previous studies. While the science behind cell-type identification was inexact, and due to difficulties in identifying correct cell types for multiple tissue samples, we annotated our results in the context of previous studies to help interpret differences and similarities between our and previous studies.

Reporting for specific materials, systems and methods

We require information from authors about some types of materials, experimental systems and methods used in many studies. Here, indicate whether each material, system or method listed is relevant to your study. If you are not sure if a list item applies to your research, read the appropriate section before selecting a response.

Materials & experimental systems

n/a	Involved in the study
☒	☐ Antibodies
☒	☐ Eukaryotic cell lines
☒	☐ Palaeontology
☒	☐ Animals and other organisms
☒	☐ Human research participants
☒	☐ Clinical data

Methods

n/a	Involved in the study
☒	☐ ChIP-seq
☒	☐ Flow cytometry
☒	☐ MRI-based neuroimaging
